# Reply to Mikhail et al. Comment on “Krysik et al. Clinical Outcomes and Early Postoperative Complications in Boston Type I Keratoprosthesis Implantation: A Retrospective Study. *J. Clin. Med*. 2024, *13*, 6710”

**DOI:** 10.3390/jcm14051614

**Published:** 2025-02-27

**Authors:** Katarzyna Krysik, Piotr Miklaszewski, Anna Maria Gadamer, Dominika Janiszewska-Bil, Anita Lyssek-Boroń, Dariusz Dobrowolski, Beniamin Oskar Grabarek, Edward Wylęgała

**Affiliations:** 1 Department of Ophthalmology, St. Barbara Hospital, Trauma Centre, 41-200 Sosnowiec, Poland; kkrysik@gmail.com (K.K.); piotrmiklaszewski94@gmail.com (P.M.); annagadamer978@gmail.com (A.M.G.); dominika.bjaniszewska@gmail.com (D.J.-B.); anitaboron3@gmail.com (A.L.-B.); dardobmd@wp.pl (D.D.); 2 Department of Ophthalmology, Faculty of Medicine, Academy of Silesia, 40-555 Katowice, Poland; 3 Collegium Medicum, WSB University, 41-300 Dabrowa Gornicza, Poland; 4 Department of Ophthalmology, District Railway Hospital, 40-760 Katowice, Poland; wylegala@gmail.com; 5 Department of Ophthalmology, Faculty of Medicine, Medical University of Silesia, 40-555 Katowice, Poland

We appreciate the insightful comments from Mikhail et al. on the results of the postoperative observations in our recent article “Clinical Outcomes and Early Postoperative Complications in Boston Type I Keratoprosthesis Implantation: A Retrospective Study” [1].

Despite careful preoperative patient selection, the most common postoperative complication after BKpro implantation is glaucoma [2,3,4]. Preexisting or secondary to keratoprosthesis implantation glaucoma in eyes with severe ocular surface diseases should be controlled as early as possible in order to maintain good visual potential [4]. But this remains a challenge even when previous procedures are applied to normalize intraocular pressure. Many patients have uncommon anatomical conditions following external valve implantation due to changes in the structure of their conjunctiva and conjunctival fornices.

Our observations of the development of glaucoma after implantation indicate a possible correlation with the formation of fibrous or amorphous membranes behind the implant. The cause may be mechanical due to the reaction to the presence of a foreign body in the anterior chamber, but it can be considered a component of uveitis.

Studies on the membranes’ structure and the expression of proinflammatory cytokines in patients with keratoprosthesis implants could explain the cause of the increased intraocular pressure. The material obtained from patients with keratoprosthesis extrusion is currently minimal and does not enable the drawing of conclusions.

We agree with the recommendation of Dr. Mikhail and colleagues that glaucoma surgery should be performed prior to or concurrently with BKPro implantation in eyes with preexisting glaucoma, in order to slow the progression of optic nerve cupping [5].

However, at present, with the current size of the group, we are not able to demonstrate all the risk factors for glaucoma recurrence or its de novo development in eyes with keratoprosthesis. A significant difficulty is the lack of an effective technique for imaging the anterior chamber and the filtration angle after keratoprosthesis implantation. The assessment of these structures, which is important for assessing the etiology of glaucoma, is exceptionally difficult.
